# Pulmonary function and medication effect in mild-stage subjects with Parkinson's disease

**DOI:** 10.1055/s-0042-1758753

**Published:** 2022-12-29

**Authors:** Renata Terra de Oliveira, Fabiana Maria dos Santos, Alessandro Gomes Ramos, Karla Luciana Magnani Seki, Paulo de Tarso Müller, Gustavo Christofoletti

**Affiliations:** 1 Universidade Federal de Mato Grosso do Sul, Faculdade de Medicina, Programa de Saúde e Desenvolvimento, Campo Grande MS, Brazil.; 2 Hospital Universitário Maria Aparecida Pedrossian, Campo Grande MS, Brazil.; 3 Universidade Federal de Mato Grosso do Sul, Instituto de Saúde, Programa em Ciências do Movimento, Campo Grande MS, Brazil.

**Keywords:** Parkinson Disease, Pulmonary Ventilation, Respiratory Function Tests, Spirometry, Oscillometry, Doença de Parkinson, Ventilação Pulmonar, Testes de Função Respiratória, Espirometria, Oscilometria

## Abstract

**Background** Parkinson's disease (PD) causes a series of movement disorders, many of them starting in the early stage.

**Objective** To analyze the pulmonary function of mild-stage subjects with PD and to investigate the effects of levodopa on it.

**Methods** We included 21 patients with idiopathic PD and 20 healthy control subjects. The participants were submitted to spirometry and impulse oscillometry assessments. The PD patients were evaluated during the “off” and “on” phases of their anti-PD medication, which was was converted to levodopa in an equivalent daily dose. A statistical analysis was performed with cross-sectional (PD patients “off” medication versus controls) and paired (PD patients “off” medication versus PD patients “on” medication) tests. The effect of levodopa was calculated with partial Eta-squared (η ^2^_ρ_ ). Significance was set at 5%.

**Results** The PD patients presented normal values in the impulse oscillometry. Regarding spirometry, the results indicated an incipient obstructive ventilatory disorder in the PD group – confirmed by patients' flow-volume curves. The PD patients received a daily dose of 354.7 ± 148.2 mg of levodopa. The paired analyses showed a small effect of anti-PD medication on pulmonary parameters (η ^2^_ρ_ = 0.197 for spirometry and η ^2^_ρ_ = 0.043 for impulse oscillometry).

**Conclusion** Patients with PD in the mild stage of the disease present pulmonary compliance and resistance compatible with normal parameters. The differences regarding the spirometric results indicate an incipient obstructive ventilatory disorder in patients with PD. Levodopa had small effect on pulmonary function in the mild stage of the disease.

## Resumo

**Antecedentes** A doença de Parkinson (DP) causa uma série de distúrbios do movimento, muitos deles se desenvolvendo na fase inicial.

**Objetivo** Analisar função pulmonar de pessoas com DP em estágio leve e investigar o efeito da levodopa nesse processo.

**Métodos** Vinte e um participantes com DP idiopática e vinte controles saudáveis foram incluídos na pesquisa. Todos os sujeitos foram submetidos a avaliações de espirometria e oscilometria de impulso. Participantes com DP foram avaliados nas fases ‘on’ e 'off' de medicação anti-parkinsoniana. A medicação dos pacientes foi convertida em dose diária de levodopa. A análise estatística foi realizada com testes transversais (Parkinson na fase ‘off’ da medicação vs controles) e pareados (Parkinson nas fases ‘off’ vs ‘on’ da medicação). O efeito da levodopa foi calculado pelo eta ao quadrado parcial (η ^2^_ρ_ ). Significância foi estipulada em 5%.

**Resultados** Pacientes com DP apresentaram valores normais na oscilometria de impulso. Na espirometria, os resultados indicaram uma desordem ventilatória obstrutiva incipiente no grupo DP – confirmada pelas curvas fluxo-volume dos pacientes. Pacientes com DP receberam uma dose diária de 354,7 ± 148,2 miligramas de levodopa. Análises pareadas mostraram baixo impacto da medicação anti-parkinsoniana nos parâmetros pulmonares (η ^2^_ρ_ = 0,197 na espirometria e η ^2^_ρ_ = 0,043 na oscilometria de impulso).

**Conclusão** Pacientes com DP na fase leve apresentam complacência e resistência pulmonares compatíveis com parâmetros normais. Diferenças espirométricas indicam distúrbio ventilatório obstrutivo incipiente em pacientes com DP. A administração de levodopa apresentou baixo efeito na função pulmonar na fase leve da doença.

## INTRODUCTION

Aging causes a series of changes that affect the proper function of the body. The increasing deposition of collagen tissue in the lung, for example, makes aging people more vulnerable to pulmonary complications. In the presence of chronic diseases, pulmonary functions tend to decline. 1 2

Parkinson's disease (PD) is one of the most common chronic neurodegenerative disorders that affect older adults. 3 4 Although movement impairment is the primary concern for patients, pulmonary problems are responsible for most causes of death. 5 6

Previous studies 7 8 9 10 have reported impairment n pulmonary function in individuals with PD. Obstructive, restrictive, and mixed patterns of respiratory dysfunction have been observed in association with reduced strength of the respiratory muscles and thoracic stiffness. 11 12

Most of the studies 13 14 describe pulmonary dysfunctions in individuals in the moderate and advanced stages of the disease, situations in which physical decline is prominent. Since age-related changes and physical decline are major causes of pulmonary dysfunction in older adults, 1 15 a question arises: are the respiratory complications in PD due to the disease itself or to an indirect effect of age or physical decline?

In a meta-analysis study, Monteiro et al. 16 showed that PD medication could have an effect on the pulmonary parameters of the subjects, indicating that treating the disease might improve pulmonary function. However, the lack of studies with mild-stage subjects and the absence of evaluations under “off-drug” conditions hinder us from ascertaining whether the onset of pulmonary problems occurs before that of physical disability. 17

The aim of the present study was to analyze the pulmonary functions and to investigate the effect of levodopa in subjects with mild-stage PD. Considering that pulmonary complications in PD are frequent in advanced stages of the disease, the authors hypothesize that mild-stage subjects would present normal parameters regarding pulmonary flow and resistance.

## METHODS

The sample consisted of 41 subjects, 21 with PD (7 women) and 20 controls (8 women). The research was conducted at the School of Medicine of Universidade Federal de Mato Grosso do Sul. All participants provided written consent prior the assessments (protocol # 3.678.458; CAAE # 22355613.0.0000.0021).

The size of the sample was calculated assuming a type-1 error of 5%, power of 90%, and an association effect of 0.474, which was extracted from a study by Sampath et al. 9 comparing pulmonary flow and resistance in PD. The analysis indicated a minimum of 38 participants, 19 in each group.

Participants with PD were recruited at the Neurologic Outpatient Clinic of the Hospital Maria Aparecida Pedrossian, in the city of Campo Grande, state of Mato Grosso do Sul, Midwestern Brazil. Subjects in the control group were selected in the community.

The PD group included patients with idiopathic PD, 18 independent for orthostatism, leading a sedentary lifestyle, and with a classification of up to stage II on the Hoehn and Yarh scale 19 during the ‘off’ phase of the medication. The control group was composed of healthy individuals matched to the PD group in terms of sociodemographic characteristics.

History of respiratory problems, thoracic alterations, and the presence of other neurologic conditions were defined as exclusion criteria for both groups. None of the participants were smokers or had been hospitalized in the twelve months preceding the study.

### Methodological procedures

The methodological procedures were reported in accordance with the Strengthening the Reporting of Observational Studies in Epidemiology (STROBE) statement. 20 The assessments were initiated with the measurement of chest wall mobility, standardized in the individual's left and right axillae. Then, the researchers used a spirometer and an impulse oscillometer previously calibrated with a 3-L syringe to measure the pulmonary parameters. All assessments were coordinated by a physician specialized in pulmonary disorders.

Chest wall mobility was calculated in centimeters with a measuring tape. The standardized procedure was to keep the part of the tape marked with zero fixed on the midline of the body, while the other end of the tape was allowed to move. The tape was positioned with a slight tightness so that the soft-tissue contours remained unchanged. For this measurement, the subjects were asked to perform maximum inspiration and expiration twice, and to hold the maximum inspiration or expiration for at least 2 s, during which the measurements were taken.

Spirometry was performed using a Koko spirometer (nSpire Health, Inc., Longmont, CO, US). The participants remained in seated in a comfortable position, and they were asked to “inflate” their lungs up to total capacity. Then, they were asked to perform maximum expiration in the device, showing at least three acceptable flow-volume curve tests for the reproduction of results. The measurements were performed according to the recommendations of the American Thoracic Society. 21 The following spirometric parameters were assessed: tidal volume (V _T_ ), forced expiratory volume in 1 s (FEV _1_ ), forced vital capacity (FVC), the FEV _1_ /FVC ratio, the FEV _1_ /V _T_ ratio, peak expiratory flow (PEF), and forced expiratory flow between 25% 75% (FEF _25-75%_ ). The values predicted for the Brazilian population were calculated according to the normality parameters established by Pereira et al. 22

The Impulse Oscillometry System (IOS, CareFusion, Höchberg, Germany) was used to assess the elastic and resistance properties of the patients' lungs. The equipment consists of a loudspeaker that works as a pulse generator that sends pressure impulses to the respiratory system. During tidal breathing, the equipment produced brief pressure pulses at intervals of 0.2 s. The superimposed pressure oscillations during normal spontaneous breathing enabled the assessment of the total volume (ToV), the central (R at 5) and peripheral (R at 20) resistances, and the pulmonary reactance (X at 5).

During the measurements, the subjects sat upright with the head in neutral position and their cheeks supported by their hands. To avoid interference of the maneuvres of spirometry with pulmonary resistance and reactance, the IOS was held before the spirometry.

All parameters were assessed during the morning. The PD group was evaluated during the “off” phase of their anti-PD medication (twelve hours after the last daily dosage), as well as during the “on” phase (one hour after the first daily dosage). The daily amount of PD medications was converted to an equivalent daily dose of levodopa (EDDL). 23

### Statistical analysis

Descriptive statistics involved the standardization of the data in mean ± standard deviation values. In the inferential analyses, we used the independent samples *t* -test to compare the PD (“off” medication) and control groups, and the paired samples *t* -test to compare the PD group n the “on” and “off” phases of the medication. The Kolmogorov-Smirnov normality test supported the use of parametric procedures. The effect size was calculated with partial Eta-squared (η ^2^_ρ_ ). Significance was set at 5%.

## RESULTS

The 41 participants were divided into the PD and control groups, which were similar in terms of sample size, age, sex, weight, height, body mass index, and chest wall mobility. The anthropometric characteristics of both groups and clinical aspects of the PD group are presented in Table 1 .

**Table 1:** Anthropometry and clinical factors of the case and control groups

Variables	Parkinson's disease group	Control group	95% Confidence interval	*p* -value
Sample size (n)	21	20	—-	0.876
Sex – male:female (n)	14:7	12:8	—-	0.658
Age (years)	70.1 ± 5.9	66.7 ± 10.1	-2.0–8.7	0.214
Weight (Kg)	70.5 ± 13.3	67.8 ± 16.3	-6.7–12.1	0.569
Height (m)	1.6 ± 0.1	1.6 ± 0.1	-0.1–0.1	0.159
Body Mass Index (Kg/m ^2^ )	26.8 ± 3.7	26.9 ± 5.6	-3.0–2.7	0.916
Chest mobility – basal (cm)	94.6 ± 6.9	91.5 ± 11.6	-3.0–9.3	0.304
Chest mobility –inspiratory (cm)	97.8 ± 7.0	96.0 ± 10.7	-3.9–7.6	0.517
Chest mobility – expiratory (cm)	93.4 ± 6.7	91.3 ± 10.2	-3.4–7.6	0.442
Hoehn and Yahr scale (points)	1.5 ± 0.4	—–	—-	—–
Daily dose of levodopa (mg)	354.7 ± 148.2	—–	—-	—–

Notes: The results are expressed as mean ± standard deviation values; *p* -value of the Chi-squared test for the categorical variables; *p* -value of the Student *t* -test for the continuous variables.

Participants' score on spirometry and impulse oscillometry are detailed in Table 2 . Comparisons between PD group (‘off’ medication) and the controls indicated differences regarding the predicted FEV _1_ , FEV _1_ /FVC ratio, FEV _1_ /V _T_ ratio, and PEF. The flow-volume curves of PD patients confirmed incipient obstructive ventilatory disorder in that group. No difference was found regarding the impulse oscillometry parameters. The comparisons of the “off” and “on” phases of the anti-PD medication indicated a mild effect of levodopa (η ^2^_ρ_ = 0.197 for spirometry and η ^2^_ρ_ = 0.043 for impulse oscillometry).

**Table 2:** Pulmonary parameters of the subjects with Parkison disease (PD, “on” and “off” medication) and of the controls

	PD “off”	PD “on”	Controls	*p* -value _(PD “off” versus control)_	*p* -value _(PD “off” versus “on”)_	Levodopa effect size
V _T_ (L)	3.7 ± 1.0	3.7 ± 1.0	3.9 ± 0.7	0.445	0.937	0.001
FEV _1_ (L)	2.7 ± 0.8	2.8 ± 0.8	2.9 ± 0.8	0.386	0.144	0.115
FEV _1_ (% of predicted)	99.1 ± 12.1	99.3 ± 13.5	111.5 ± 19.9	0.022	0.861	0.027
FVC (L)	3.4 ± 0.9	3.5 ± 0.9	3.5 ± 0.7	0.588	0.146	0.081
FVC (% of predicted)	103.8 ± 13.0	105.8 ± 11.5	111.3 ± 14.7	0.092	0.175	0.029
FEV _1_ /FVC (%)	77.3 ± 8.2	77.6 ± 7.3	81.7 ± 4.4	0.041	0.761	0.070
FEV _1_ /V _T_ (%)	75.4 ± 6,8	75.9 ± 5.2	81.4 ± 6,0	0.005	0.532	0.197
PEF (L/s)	5.4 ± 1.8	5.3 ± 1.6	7.2 ± 1.0	0.001	0.632	0.001
FEF _25-75%_ (L/s)	2.3 ± 1.0	2.3 ± 1.0	2.6 ± 0.6	0.270	0.991	0.001
ToV (L)	0.9 ± 0.3	1.0 ± 0.3	0.9 ± 0.3	0.941	0.793	0.009
R at 5 (cmH _2_ O/L/s)	3.2 ± 1.0	3.2 ± 1.1	3.3 ± 0.7	0.691	0.759	0.004
R at 20 (cmH _2_ O/L/s)	2.7 ± 0.7	2.6 ± 0.7	2.8 ± 0.8	0.518	0.562	0.043
X at 5 (cmH _2_ O/L/s)	-1.1 ± 0.3	-1.1 ± 0.3	-1.3 ± 0.3	0.323	0.493	0.008

Abbreviations: FEF _25-75%_ , forced expiratory flow; FEV _1_ , forced expiratory volume in 1 second; FVC, forced vital capacity; PEF, peak expiratory flow; ToV, total volume; V _T_ , tidal volume.

Notes: R at 5, central resistance; R at 20, peripheral resistance; X at 5, pulmonary reactance; *p* -value of the independent samples *t* -test (PD “off” medication versus controls); *p* -value of the paired samples *t* -test (PD “off” versus “on” medication).

## DISCUSSION

The main objective of the present study was to analyze pulmonary function in mild-stage PD patients. The results showed normal parameters for lung compliance and resistance in the PD group. Despite the normal values on impulse oscillometry, the spirometric scores indicate an incipient obstructive disorder in patients with PD. We will now discuss the results, and we believe our findings may be of interest to patients, families, researchers and health care professionals.

In the present study, we used the Hoehn and Yahr scale to define mild-stage PD (score of up to II on the “off” phase of medication). Several other instruments, such as the Movement Disorder Society-sponsored revision of the Unified Parkinson Disease Rating Scale, 24 the Parkinson Disease Dyskinesia Scale, 25 and the Clinical Global Impression Scale, 26 may also be used to classify PD severity. We opted to use the Hoehn and Yahr scale because it is one of the most frequently used scales for the global assessment for PD. 27

Impairment of pulmonary function has been demonstrated in individuals with PD, especially when subjects are on the moderate or severe stages of the disease. 7 8 9 10 11 12 There are several mechanisms that explain respiratory problems in PD. For instance, by being directly involved in the disease, some mechanisms enable the classification of pulmonary dysfunctions as a symptom of PD. Others, more related to complications arising from the physical decline, tend to classify the pulmonary dysfunctions as an indirect effect of PD.

Physical decline starts in the early stages of the disease. 28 29 However, it is in more advanced stages that mobility is more affected, and the rate of homebound patients increases. 30 As pulmonary dysfunction is common in advanced PD, we have hypothesized that these disorders might be more related to the impairments in mobility and their complications rather than been a direct effect of PD.

The results of impulse oscillometry demonstrated that pulmonary compliance (X at 5) and resistance (R at 5 and at 20) in the early stage of the disease are compatible with normal parameters. Regarding spirometry, we observed the early stages of obstructive disorder in patients with PD. This is explained by the normal parameters found for FEV _1_ (higher than 80% of the predicted value) and the low values found for FEV _1_ /FVC and PEF (below 80%, which is the minimum accepted predictive parameter).

Despite the limitation of cross-sectional studies to establish cause–effect relationships, the results of the present study are important, as they indicate an incipient pulmonary disorder in patients with PD, even among the subjects who did not report previous respiratory illness or symptoms. Further studies with longitudinal assessments should be carried out for a more precise analysis of the impact of disease severity on patients' pulmonary functions.

The role of levodopa in respiratory function has been under debate for years. Some studies 11 16 have described it as having an adverse dopaminergic effect. Others have reported that pulmonary parameters have been improved after levodopa administration. 16 31 32 Considering the influence of levodopa on respiratory function, we opted to evaluate PD subjects in the “off” and “on” phases, isolating possible biases caused by the medication.

Nonetheless, we did not find any significant effect of the anti-PD medication on patients' pulmonary function. The effect size analyses, in fact, indicated a mild impact on spirometry and impulse oscillometry ( Table 2 ).

The absence of significant effects of the medication, however, must be interpreted with caution. It is likely that the lack of interference of levodopa with the results could be due to the profile of the sample, which was composed of participants without significant pulmonary disabilities. Thus, the findings do not contradict the studies 16 31 32 that point out the effects caused by the anti-PD medication on pulmonary function, but they show that, in individuals in the mild stage of the disease, the effect of levodopa on the respiratory system is not significant.

### Limitations

The present study has two important limitations. The first one refers to the description of the clinical condition of the patients. As aforementioned, we only used the Hoehn and Yahr scale to characterize the mild stage of the disease. Further studies seeking to perform complementary analyses of the impact of disease severity on subjects' pulmonary function should detail the clinical conditions of a larger number of patients. The second limitation refers to the small sample size. We included only 21 individuals with PD. It is worthy of note that recruiting subjects in the initial stage is challenging because the diagnosis is difficult, and much of the symptoms are not noticeable.

In conclusion, pulmonary compliance and resistance in mild-stage PD patients are compatible with normal parameters. The spirometric measurements indicate an incipient obstructive disorder in the PD group. The administration of levodopa had a mild effect on the pulmonary functions of patients in the early stage of the disease.
